# Power and chirp effects on the frequency stability of resonant dispersive waves generated in photonic crystal fibres

**DOI:** 10.1038/s41598-017-18544-y

**Published:** 2018-01-09

**Authors:** Tao Cao, Mingchen Liu, Chang Xu, Jikun Yan, Chaochao Shen, Shaozhen Liu, Hao Peng, Jiahui Peng, Alexei V. Sokolov

**Affiliations:** 1 School of Optical and Electronic Information, Huazhong University of Science and Technology, Wuhan, 430074 China; 2 Department of Physics and Institute for Quantum Studies, Texas A&M University, College Station, Texas, 77843-4242 USA

## Abstract

Optimization of laser output parameters vs. f-to-2f beating signals can be mutually contradicting, when an octave-spanning supercontinuum is employed for f-to-2f measurements. We show that resonant dispersive waves will solve this issue, thanks to their frequency stability against changes in laser power and chirping.

## Introduction

The so-called f-to-2f method, which is used to measure carrier-envelope offset (CEO) frequency, is among the key techniques of optical frequency combs, which have had significant impact on high-precision optical frequency metrology, optical clocks, low-phase-noise microwave generation and many other fields^1–4^. Typically, in f-to-2f techniques, a higher-frequency component of the octave-spanning supercontinuum is made to interfere with the second harmonic generated by the corresponding lower-frequency spectral component, and the radio-frequency beating signal indicates the CEO frequency^1–3,5–10^.

Once the f-to-2f setup is in place, changing the output power or chirp of the laser pulses becomes problematic as it will lead to dramatic changes in the supercontinuum spectral envelope^11^. Given the nature of f-to-2f techniques, these changes in spectral envelope can degrade the beating signal, and thus the CEO measurement, significantly, because the two corresponding frequency components employed in the f-to-2f technique will not necessarily remain as intense. That is, the output laser properties and the f-to-2f measurement can hardly be optimized simultaneously. This is especially true for fibre lasers, where achieving precise dispersion control is difficult because it is impossible to cleave-and-splice fibres to exact lengths every time. Even when great efforts are made to optimize laser output power and pulse duration, in reality, fibres often need to be re-spliced when undergoing maintenance. In those cases, the output laser properties are always compromised to obtain better, if not the best, beating signals.

It has been recently found that resonant dispersive wave (RDW) generation can be used to produce tunable femtosecond pulses with quite good efficiencies and can be much more flexibly applied to measure CEO frequencies for tunable femtosecond lasers^12^. In this paper, we present experimental results showing that spectral envelopes of RDWs are comparably stable against changes in both laser power and chirping, which makes RDWs a good route for solving the abovementioned issue.

## Results and Discussion

When there is a perturbation acting on a soliton (e.g., by a localized loss in the fibre or modified parameters in the laser cavity), a soliton will reshape its form and shed the excess energy into RDWs. This process can be understood as soliton fissions accompanied by soliton Cherenkov radiation^11–17^, and the frequencies of the RDWs (*ω*) are mainly determined by the phase-matching conditions^18,19^

1 $$\sum _{m\mathrm{=2}}^{\infty }\frac{{k}_{m}({\omega }_{sol})}{m!}{(\omega -{\omega }_{sol})}^{m}=\frac{1}{2}\gamma {P}_{sol},$$

where *k*_*m*_ = ${d}^{m}k/d{\omega }^{m}$ (*k* is the frequency-dependent wavenumber), *ω*_*sol*_ and *P*_*sol*_ are the centre angular frequency and the peak power of the radiative soliton, respectively, and *γ* is a nonlinear coefficient.

**Figure 1:**
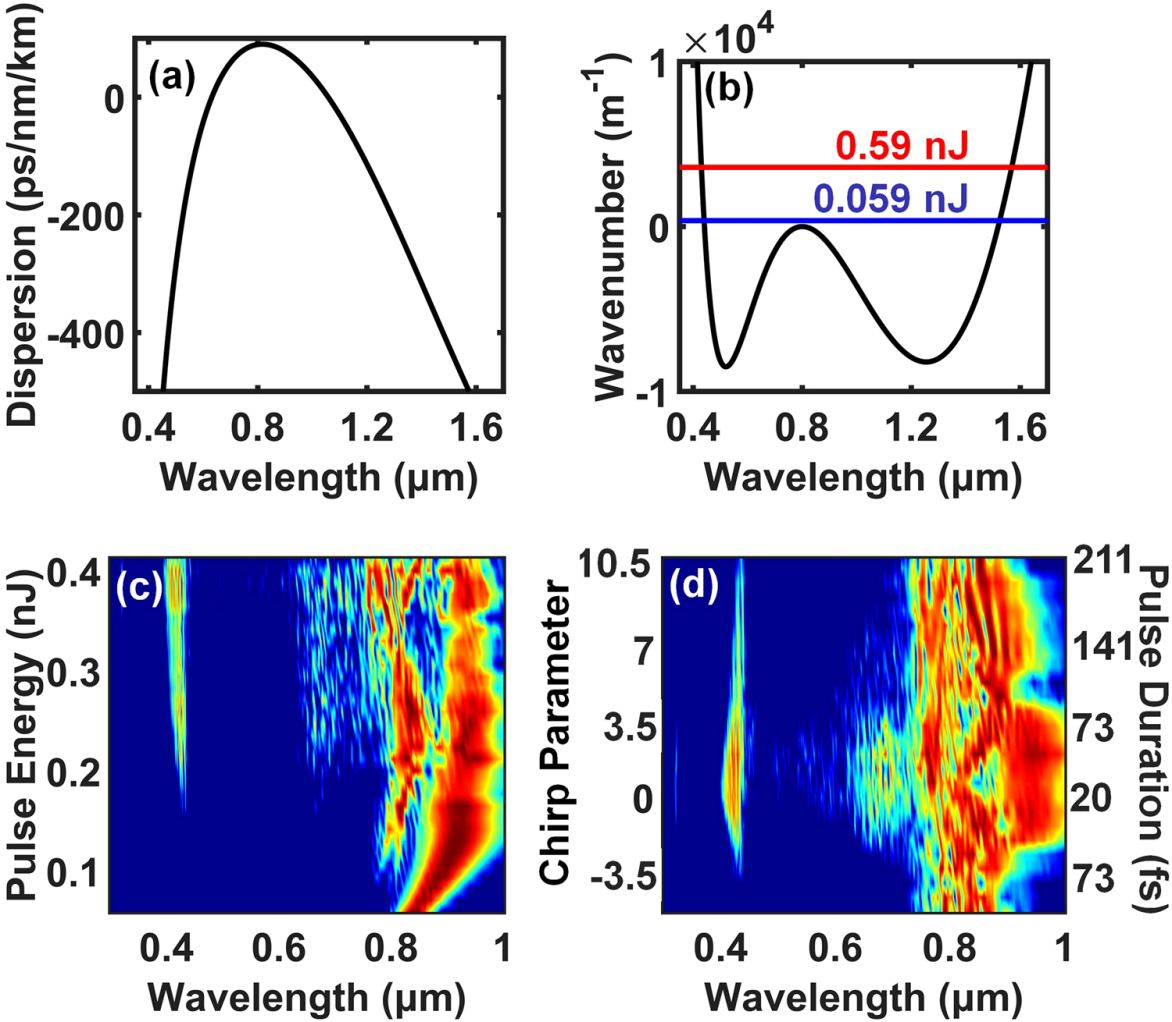
(**a**) Calculated GVD curve of one of a cladding node of the Kagome PCF. (**b**) Phase-matching curve. The lower line (0.059 nJ pulse energy) and the upper line (0.59 nJ pulse energy) intersect with the dispersion curve at the phase-matching wavelengths of 440 nm and 430 nm, respectively. (**c**) Simulation results of output spectra at different input pulse energies with an unchirped pump pulse. (**d**) Simulation results of output spectra at different chirp parameters with an input pulse energy of 0.28 nJ. The duration of the unchirped pulse is 20 fs. The intensity is normalized to the maximum value for each input pulse energy or chirp parameter.

The phase-matching conditions for a 20 fs transform-limited (TL) Gaussian pulse at a wavelength of 800 nm that is propagating through a fibre with a nonlinear coefficient *γ* = 0.13 W^−1^ m^−1^ and group velocity dispersion (GVD) properties shown in Fig. 1(a) are described in Fig. 1(b). Figure 1(b) clearly shows that the phase-matched wavelength of the RDW only changed by 10 nm, even though the input energy increased by ten times. It is this nature that makes RDWs stable against changes in pump laser power, chirping, or both. In fact, any fibre with a dispersion curve that is sufficiently steep at the phase-matched wavelength (as shown in Fig. 1(b)), and with a sufficiently high nonlinear coefficient, can be used to achieve the stability. In the experiments described below, RDW generation is realized in selected cladding nodes of a Kagome PCF, which possesses the characteristics described above.

In support of this argument, we have conducted simulations using the split-step Fourier method to numerically solve the generalized nonlinear Schrödinger equation (GNLSE) under the conditions described above and with a fractional contribution of the Raman nonlinearity to the Kerr nonlinearity of *f*_*R*_ = 0.18. The simulated output spectra are shown in Fig. 1(c) and (d) for varying energies and chirp parameters, respectively. The results clearly show that the envelopes of the RDW’s spectra change much less dramatically than those of the supercontinuum, when the input power and chirp are changed. In addition, it is worth noting that the excitation of the RDWs is rapidly suppressed with the increase in negative chirp, as shown in Fig. 1(d), which confirms that RDW generation is strongly correlated to solitons.

**Figure 2:**
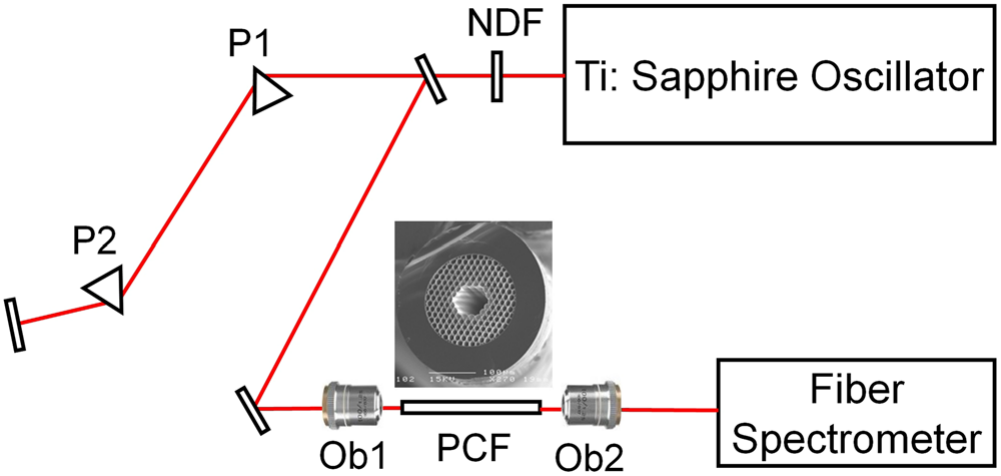
Experimental setup: NDF is a neutral density filter; P1 and P2 are prisms; Ob1 and Ob2 are 20× objective lenses. The STM image of the PCF cross section is shown in the inset.

Our experiments, described below, are consistent with the above simulation results. The experimental setup is shown in Fig. 2. In these experiments, an 85 MHz Ti:Sapphire oscillator (KMLabs, TS laser kit) and a 9-cm long Kagome PCF were used to generate RDWs. A neutral density filter was used to adjust the laser power, and a four-prism system (outside the oscillator cavity) was employed to adjust the pulse duration by adjusting the chirp introduced to the laser pulses. The pulse durations reported below were measured after the coupling lens using an autocorrelator (APE, pulse check) to obtain the intensity full-width-at-half-maximum (FWHM) pulse duration under a Gaussian pulse shape assumption.

**Figure 3:**
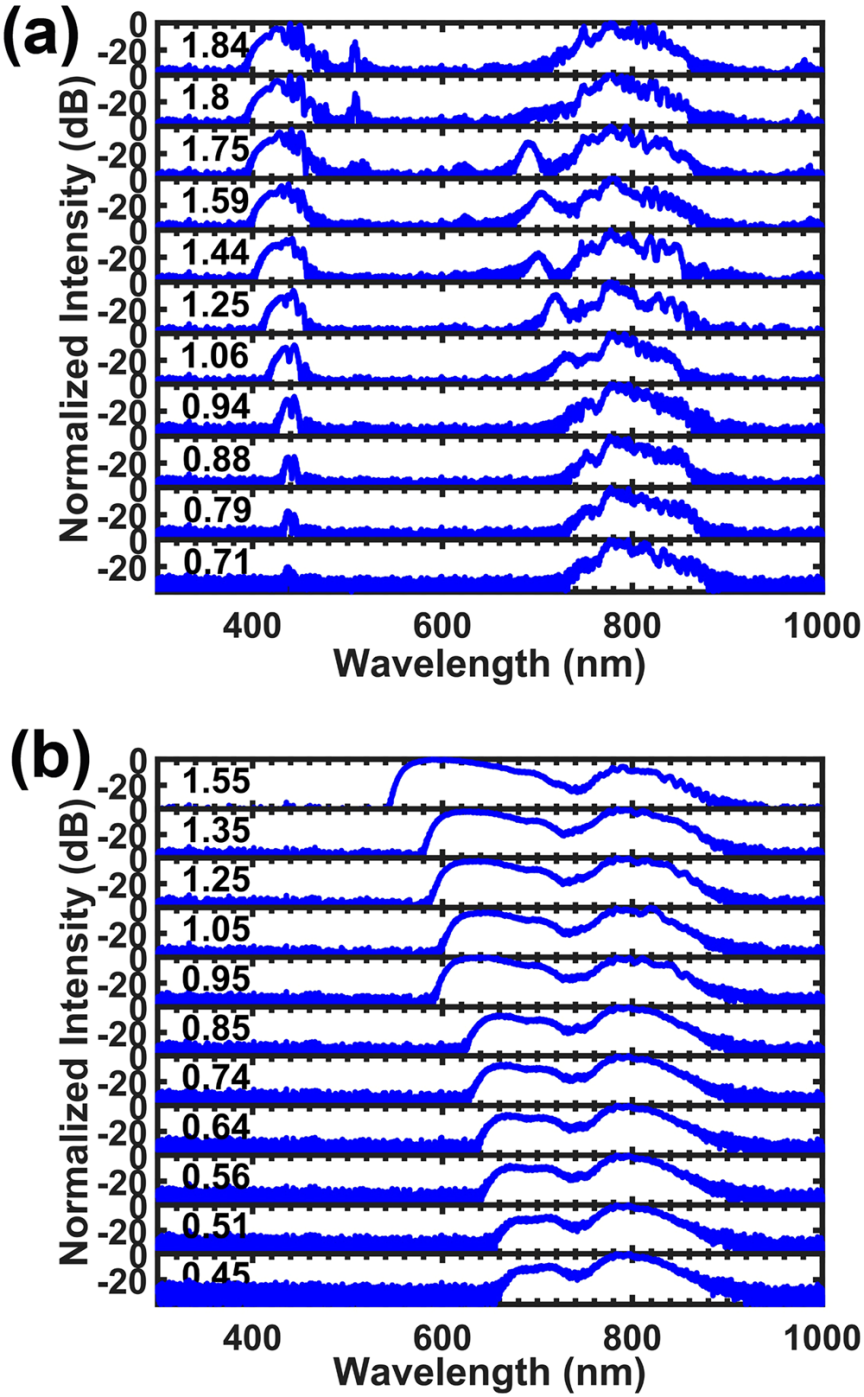
Experimental output spectra for different input energies (**a**) with excitation of RDWs and (**b**) without excitation of RDWs, with pump pulse spectra centred at 800 nm. The energies before coupling are labelled on the left side of each spectrum in units of nJ (the coupling efficiency is ~10%). The pulse durations were all 20 fs.

The RDW spectra generated by 20 fs optical pulses with centre wavelengths of 800 nm at different input pulse energies are shown in Fig. 3(a). In addition to the spectral broadening at ~800 nm due to self-phase modulation (SPM), soliton fissions and Raman effects, the spectra show isolated peaks localized at approximately 440 nm due to RDW generation. The unusually large peak power values of the RDWs indicate strong energy transfers from the soliton body to the RDWs. In addition to this efficient energy conversion, it is important to note that the centre wavelength of the RDWs remained almost unchanged (the change was less than 5 nm), even though the input energy (before coupling) changed from 1.84 nJ to 0.71 nJ, which was consistent with the simulations as well.

As mentioned above, the frequency stability of RDWs is quite helpful for f-to-2f measurements, whereas a supercontinuum would not have that capability. For comparison, the experiments described above were repeated without efficient excitation of RDWs. This was achieved by translating the fibre transversally within one micron of its original position. Under that condition, more of the input energy was transferred to blueshifted frequencies via SPM, with the maximum value of the frequency shift estimated by

2 $$\delta {\omega }_{max}\approx {n}_{2}\frac{{\omega }_{0}}{{T}_{0}c}{I}_{0}L,$$

where *n*_2_ is the Kerr coefficient, *c* is the speed of light, *L* is the fibre length, *ω*_0_ is the carrier frequency of the initial pulse, *T*_0_ is the duration of the initial pulse, and *I*_0_ is the peak intensity of the initial pulse^20^. Equation (2) indicates that the frequency shift is directly proportional to laser intensity, which is confirmed by our experimental results, as shown in Fig. 3(b). These results clearly show that changing the laser power will change the spectral envelope and thus degrade the beating signal, because the peak-power frequency shifts with input power if the SPM-induced segment of the supercontinuum is used, which is common in f-to-2f measurements.

Fibre lasers are superior to other solid-state lasers because of their stability and freedom from maintenance that arises from the fact that all of their components are simply spliced together. However, the output supercontinuum spectra changing with input laser power can become a problem when working with fibre lasers. In addition, since it is impossible to keep all fibre components in every fibre laser at exactly the same length after being spliced together, the dispersion parameters vary from one laser to another. This reality means that output laser power and pulse duration of femtosecond fibre lasers vary slightly from one laser to another because both gain and pulse duration are strongly correlated with dispersion. Moreover, the lengths of the fibre components will change after service is performed, and output power and pulse duration will then also change.

Outside of f-to-2f technique related applications, this may not be a big issue. For the f-to-2f technique, however, the frequency doubling crystal is chosen for fixed phase-matching conditions, either by setting the orientation of the nonlinear crystal or by selecting the pattern of quasi-phase-matching structures. To obtain a good f-to-2f beating signal, there must be sufficient power in the spectral regions of both the fundamental and second harmonic frequencies of the supercontinuum output. However, any variation in the laser output parameters will cause dramatic changes to the supercontinuum spectra. Because of the loss of freedom when choosing frequency components for f-to-2f measurements, it becomes impossible to optimize both the laser output parameters and the f-to-2f measurements simultaneously. In reality, laser output is sacrificed to optimize f-to-2f measurements. Our demonstrated frequency stability of RDWs will eliminate these limitations and allow to simultaneously optimize laser output parameters and f-to-2f measurements.

**Figure 4:**
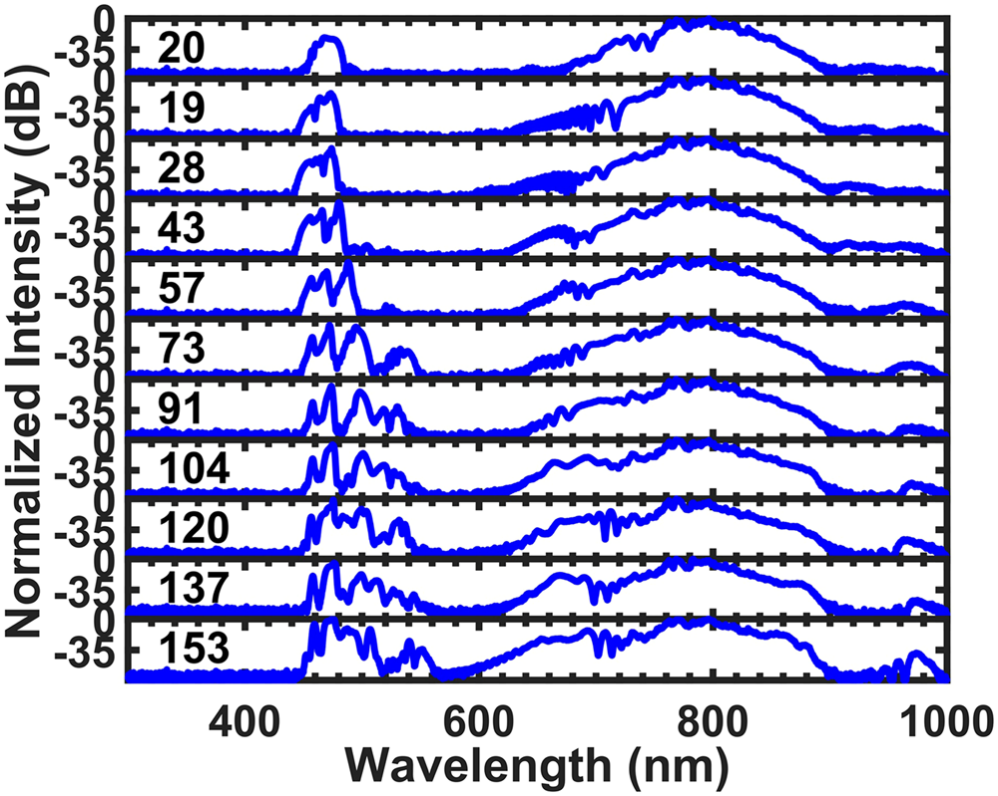
Experimental output spectra results for different chirps for pump pulse spectra centred at 800 nm. The durations of the chirped input pulses are marked on the left side of each spectrum in units of fs. The pulse energies were all 2.35 nJ (the coupling efficiency is ~10%).

In addition to power instability, pulse duration is a parameter whose instability can cause dramatic changes in the output spectra^11^. Again, the pulse duration is not an adjustable parameter in an all-fibre femtosecond laser, and RDWs will result in better performances vis-á-vis this issue as well. In the experiments discussed below, the pump pulse energies were fixed at 2.35 nJ (before coupling), but the pulse durations were varied. We started with 20 fs pulses, which were slightly negatively-chirped (with a chirp parameter of −0.33), and then gradually introduced positive chirp to the input pulses by inserting one of the external prisms deeper into the laser beam. The output spectra, after the PCF, are shown in Fig. 4. Even though the durations of the input chirped laser pulses changes from 19 fs to 153 fs, which corresponds to the chirp parameter changing from 0 to 7.99, the centre wavelength of the RDW remains nearly unchanged.

Unlike the previous case, these RDW spectra exhibit more features, and new RDW wavelengths appeared with the stretched pulses. This is not surprising, given that the generation of RDWs is a solitonic behaviour. As pulse duration increases from 19 fs to 153 fs, the order of the formed soliton, the soliton number, will increase, and fissions of higher-order solitons into fundamental solitons will excite more RDWs in different frequency regions. In addition, as the soliton number increases, coherence of the generated RDWs degrades because of modulation instability^11,21,22^, which might degrade the f-to-2f beating signal until it totally disappears. Therefore, even though the f-to-2f beating signal obtained by our methods is less sensitive to changes in pump pulse chirp, the pulse duration should be kept short enough to maintain RDW’s coherence.

We also tried to stretch the input pulses by introducing negative chirp. The generation of RDWs declined until completely disappearing, in agreement with our simulation results. Given that no solitons can be formed when too much negative chirp is introduced, these results again reveal the relationships between RDWs and solitons.

## Conclusions

In conclusion, the simulation and experimental results show that RDWs generated in a PCF have robust frequency stabilities against changes in laser powers and chirping. This behaviour reveals that output laser properties and f-to-2f beating signals can be jointly optimized without sacrificing much of the laser performances if RDWs are applied in f-to-2f measurements^12^. This is expected to be especially useful for all-fibre frequency comb sources. Although the laser employed in our experiments was a solid-state laser rather than a fibre laser (because of the type of PCF presently available to us), these proof-of-principle results would not change with laser type.
